# Zonulin as Gatekeeper in Gut–Brain Axis: Dysregulation in Glioblastoma

**DOI:** 10.3390/biomedicines12081649

**Published:** 2024-07-24

**Authors:** Hannah Hagemeyer, Olaf J. C. Hellwinkel, Julio Plata-Bello

**Affiliations:** 1 Institut für Neuroimmunologie und Multiple Sklerose, University Medical Center Hamburg-Eppendorf, Falkenried 94, 20251 Hamburg, Germany; hannah.hagemeyer@zmnh.uni-hamburg.de; 2 Department of Forensic Medicine, University Medical Center Hamburg-Eppendorf, Martinistraße 52, 20251 Hamburg, Germany; 3 Department of Neurosurgery, Hospital Universitario de Canarias, S/C de Tenerife, 38320 La Laguna, Spain

**Keywords:** blood–brain barrier, glioblastoma, zonulin, pre-haptoglobin

## Abstract

Novel biomarkers and therapeutic strategies for glioblastoma, the most common malignant brain tumor with an extremely unfavorable prognosis, are urgently needed. Recent studies revealed a significant upregulation of the protein zonulin in glioblastoma, which correlates with patient survival. Originally identified as pre-haptoglobin-2, zonulin modulates both the intestinal barrier and the blood–brain barrier by disassembling tight junctions. An association of zonulin with various neuroinflammatory diseases has been observed. It can be suggested that zonulin links a putative impairment of the gut–brain barrier with glioblastoma carcinogenesis, leading to an interaction of the gut microbiome, the immune system, and glioblastoma. We therefore propose three interconnected hypotheses: (I) elevated levels of zonulin in glioblastoma contribute to its aggressiveness; (II) upregulated (serum-) zonulin increases the permeability of the microbiota–gut–brain barrier; and (III) this creates a carcinogenic and immunosuppressive microenvironment preventing the host from an effective antitumor response. The role of zonulin in glioblastoma highlights a promising field of research that could yield diagnostic and therapeutic options for glioblastoma patients and other diseases with a disturbed microbiota–gut–brain barrier.

## 1. Introduction

Glioblastoma is the most common malignant brain tumor, but also the brain tumor with the lowest median survival of only 8 months and thus the major reason for death from primary cerebral neoplasms [1]. According to the classification delineated by the World Health Organization (WHO), glioblastomas are defined as IDH-wildtype gliomas [2]. Histologically, glioblastomas are characterized by an elevated cell density with high mitotic activity, atypical cell types including hyperchromatic and pleomorphic nuclei, extensive necrotic regions, and robust neovascularization [3,4]. The standard treatment entails maximal safe surgical excision while preserving neurological functionality, followed by concurrent administration of radiation and the DNA alkylating agent temozolomide [5]. Despite ongoing research and these triple therapeutic options, the progression-free survival ranges only from 1.5 to 4.2 months [6], which has not significantly increased within the last years. Merely 6.9% of patients survive beyond five years [1,7,8]. These limited diagnostic and therapeutic options emphasize the urgent need for research in the field of glioblastomas to ameliorate the prognostic outlook.

Recent studies have unveiled a significant upregulation of the protein zonulin exclusively in glioblastoma, correlating negatively with patient survival [9]. Originally identified as pre-haptoglobin-2, zonulin modulates intestinal and blood–brain barrier function through the disassembly of tight junctions [10]. Lately, zonulin has attracted growing attention as it shapes several neuroinflammatory diseases such as Major depressive disorders [11], Alzheimer’s disease [12], Multiple Sclerosis [13,14], Myalgic encephalomyelitis/Chronic fatigue syndrome (ME/CFS) [15], Chronic fatigue syndrome in schizophrenia [15], schizophrenia [16], and Parkinson’s disease [17,18,19].

Zonulin represents a potential link between a leaky gut–brain axis and glioblastoma. The interplay of the immune system, the gut microbiome, and glial tumor carcinogenesis is influenced by zonulin. The gut–brain axis (GBA) represents a bidirectional exchange platform between the gastrointestinal tract and the central nervous system (CNS) dedicated to maintaining homeostasis between environmental and internal signals [20]. Based on heightened zonulin production in glioblastoma, three hypotheses can be proposed, potentially enhancing each other in a vicious cycle: (I) zonulin contributes to tumor aggressiveness; (II) the elevated zonulin levels in glioblastoma and the corresponding blood-stream increase the permeability of the microbiota–gut–brain axis; and (III) higher trafficking of microbiota and immune cells subsequently induces a chronic inflammatory and carcinogenic microenvironment. A local immunosuppression impairs the hosts to eliminate glial tumor cells. In this review article, we will endeavor to provide the justifications supporting these hypotheses in the intricate interplay of zonulin and glioblastoma.

## 2. Zonulin: Regulating Intestinal Barrier Permeability and Beyond

**Figure 1:**
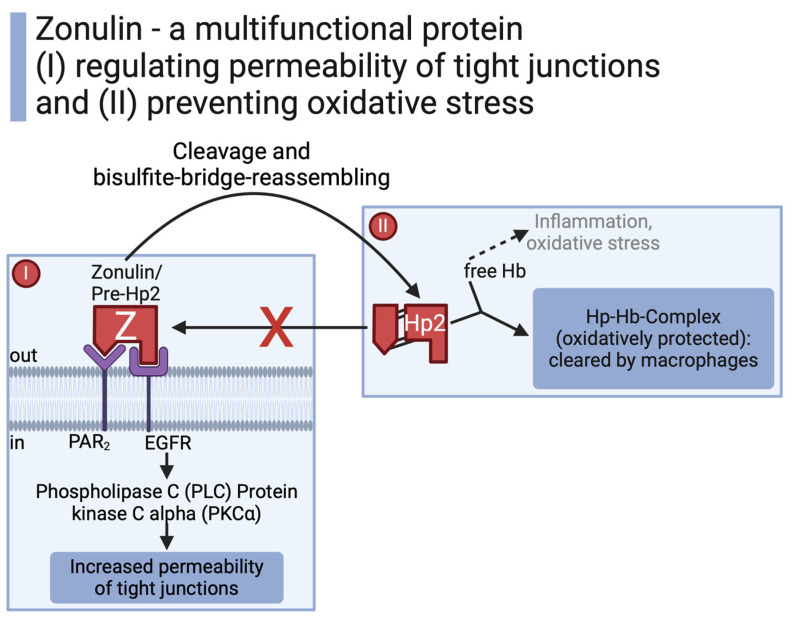
While zonulin (Pre-Hp2) increases permeability of tight junctions, its “mature” form Hp2 prevents oxidative stress [10,14,21,22,23]. Created with BioRender.com.

Through what started off as a failed attempt for a vaccination against cholera [21], the discovery of zonulin revolutionized the view of the microbiota–gut–brain axis. Zonulin corresponds to the precursor of haptoglobin-2 (Hp2) [14]. As a multifunctional protein, zonulin can either act in its uncleaved form to regulate the permeability of tight junctions or (after cleavage and bisulfite bridge reassembling) as “mature” Hp2 to build a haptoglobin–hemoglobin complex preventing tissue damage by oxidative stress [14]. Upon formation of mature Hp2, the protein loses its function to induce paracellular permeability (Figure 1) [22]. 

The uncleaved 47 kDa human protein zonulin was originally identified as a protein mimicked by zonula occludens toxin (Zot), an enterotoxin by Vibrio cholerae modulating the intestinal barrier [23,24]. The bacterial enterotoxin and the endogenous human zonulin act through the same intracellular pathway to regulate the permeability of intestinal tight junctions [14,23,25]. Tight junctions do not merely consist of the bare link between two cells but are rather a complex network of transmembrane proteins, peripheral membrane proteins, and intracellular regulatory factors [26]. Mechanistically, zonulin transactivates the epidermal growth factor (EGFR) directly or via proteinase-activated receptor 2 (PAR_2_). This induces an intracellular cascade of phospholipase C (PLC) and protein kinase C alpha (PKCα) [10,22,23]. 

EGFR and PAR_2_ receptors are expressed on brain endothelial cells and small intestinal epithelial cells. Therefore, zonulin is involved in regulating the segregation of—firstly—pathogens from host tissue in the gut and—secondly—immune cells from the “immune-privileged” CNS [10]. Besides being synthesized in the small intestine and liver, zonulin can be detected in the brain, heart, lungs, adipose tissue, immune cells, kidneys, and skin [23,27,28]. The gut immune barrier, consisting of (entodermal-) epithelial cells, segregates the gut lumen from the host internal space. In contrast, the blood–brain barrier (BBB), composed of (vaso-) endothelial cells, pericytes, and astrocytes, isolates the CNS parenchyma from the lumen of blood vessels [29]. Compared to the leakiness of blood vessels in most human tissue, trafficking of immune cells and pathogens across the BBB is tightly restricted to protect the CNS [29]. Taken together, zonulin shapes the permeability of the intestinal barrier and the BBB. 

As the largest interface between the human organism and the environment, the intestinal barrier has to be tightly regulated to prevent pathogen invasion and to flush out microorganisms that already entered the host. Nonetheless, a sufficient nutrient and electrolyte exchange with digested food in the gut is necessary [14,30]. Due to its regulatory function in intestinal epithelial tight junctions [10,31], zonulin plays a crucial role in the control of the gut immune barrier in diseases such as Celiac disease [24] and inflammatory bowel disease [27]. Different stimuli trigger zonulin release in the intestinal epithelium. As a component of gluten in wheat, gliadin induces zonulin release and increases the permeability of the intestinal barrier, which is a postulated mechanism in the pathogenesis of Celiac disease (CD) [32,33]. Another main trigger for intestinal zonulin secretion involves several pathogenic and non-pathogenic enterobacteria such as *E. coli* and *Salmonella typhy* [34]. The high amino acid sequence identity (90%) of gliadin and bacterial proteins may explain their shared ability to induce zonulin release through binding to C-X-C chemokine receptor type 3 (CXCR3) [35]. Elevated zonulin levels are associated with pathogen overgrowth in stool samples [36]. However, zonulin can also flush invading microorganisms back into the intestinal lumen [34], and some microbiota such as *Lactobacillus* even counteract a disruption of the epithelial barrier by preventing zonulin release [37]. In summary, both gliadin and intestinal bacteria can stimulate zonulin release and subsequently increase tight junction permeability.

## 3. Glioblastoma, Gut Microbiome, and Immune Microenvironment—Interconnected in Tumor Progression and Therapeutic Response

Glioblastomas induce a proangiogenic, inflammatory microenvironment and a more permeable BBB [38]. The compromised barrier function is clinically observed through enhanced gadolinium contrast medium and intense vasogenic edema surrounding glioblastoma in magnetic resonance imaging [9]. The leaky BBB facilitates the invasion of microbiota and immune cells normally absent from the “immune-privileged” CNS [38,39,40,41]. For example, bacterial DNA has been detected in glioblastoma samples [42,43]. The relationship between glioblastoma and the gut microbiome appears bidirectional, as glioblastoma seems to influence the gut microbiome composition [44], while a dysbiosis of the gut microbiome could shape glial tumor growth [45]. 

The load of certain microbiota was increased in glioblastoma mice and patients compared to healthy controls, which was neutralized under temozolomide treatment [44]. Microbiota have also been reported to influence tumor outcome directly by modifying inflammatory transcriptional pathways, tumor carcinogenesis, and tumor-infiltrating immune cells [46]. As a key factor in glial tumor progression, gut microbiota modify neurotransmitters and subsequently influence glioma cell proliferation [47]. Infiltrating immune cells sensitized to pathogenic or commensal bacterial peptides can cross-recognize tumor antigen-derived peptides presented by HLA molecules on glial tumor cells [48]. Consequently, intra-tumoral bacteria may modulate the antitumoral immunological response [48]. Heterogenic opinions exist regarding concrete agents of the microbiome related to gliomas. For instance, gliomas have been associated with increased levels of *Verrucomicrobia*, *Akkermansia*, *Intestinimonas*, and *Lactobacillus* but decreased levels of *Anaerotruncus* [44,49]. Both increased and decreased levels of *Firmicutes* and *Bacteroidia* have been described in the literature [44,45]. These microorganisms can modulate tumor development: coadministration of *Bifidobacterium lactis* with *Lactobacillus plantarum* [50] and compound **K** (a metabolite of ginseng produced by intestinal bacteria [51]) could reduce glioma cell migration and/or proliferation in vitro. In contrast, chronic antibiotic treatment of glioma-bearing mice leads to a higher glial tumor growth with reduced cytotoxic natural killer (NK) cell subsets [52]. Taken together, a disbalanced gut microbiome and glioblastoma seem to influence—and partially enhance—each other bidirectionally. 

This interconnection raises the question as to whether the immunosuppressive microenvironment of glioblastoma provides a refuge for locally invading microbiota (favorable for glioblastoma development) without causing a severe cerebral infection [53]. A possible explanation for the intracranial immunosuppressive milieu surrounding glioblastoma [54,55] could be immune exhaustion as a prolonged and insufficient response to chronic pathogen invasion across the BBB. A substantial influx of microglia and tumor-associated macrophages (TAMs) infiltrates glial tumors, compromising approximately 30% of viable cells within glioblastoma [56,57]. While microglia traditionally regulate immune responses in the brain, they fail to launch an effective antitumor response in glioblastoma. Microglia have come under control of the tumor and exert immunosuppressive functions leading to a predominance of Th2 cytokines [58,59,60]. Immunosuppressive cytokines such as Transforming growth factor-β2 (TGF-β2), Prostaglandin E_2_ (PGE_2_), and Interleukine-10 (IL-10) within glioblastoma limit phagocytic activity and induce apoptosis in activated lymphocytes [54,61,62]. Elevated levels of these immunosuppressive mediators correlate with glial tumor grade: high-grade gliomas express elevated IL-10 and TGF-β levels [63,64]. TGF-β also downregulates the activating receptor NKG2D on NK cells and CD8-T cells, impairing their efficiency in killing mutated or infected target cells [65]. Altogether, glioblastomas can control infiltrating microglia and tumor-associated macrophages (TAMs) in favor of an immunosuppressive microenvironment.

The source of immunomodulatory cytokines in response to invading microbiota remains unclear, with potential contributors including glioma cells themselves, M2 monocytes, or regulatory T cells (T_regs_) [66,67,68]. Glioma cells secrete cytokines that restrict Major Histocompatibility Complex class II (MHCII) production in microglia. The subsequently limited antigen-presenting capability prevents cytotoxic T cell activation against tumor cells [69]. The glioma-shaped microglia also induce T cell apoptosis through FasL and B7-H1-mediated apoptosis [70,71]. While the restriction of immune responses normally serves as a negative feedback mechanism to reduce an overshooting CNS inflammation after pathogen invasion or prevent autoimmunity, glioma cells hijack this mechanism to evade immune surveillance [72]. Invading microbiota and modified immune cells establish local immunosuppression, which presents a major challenge in the development of immune therapies for glioblastoma patients [72,73,74]. For instance, trials with anti-PD-1 antibodies failed to demonstrate prognostic advantages in glioblastoma [75,76,77]. Even though the BBB is more permeable in glioblastoma, metastases outside of the CNS are very rare. This restriction to the CNS might be explained through local immunosuppression around glioblastoma and prevention of extracranial spread through a still effective systemic immune response with the glioblastoma-characteristic immunosuppressive factors being absent in systemic circulation [62,78]. Glioblastomas escape from the host immune system through local immunosuppressive milieu.

## 4. Hypotheses: Zonulin as Dysregulated Gatekeeper of the Microbiota–Gut–Brain Axis in Glioblastoma

Three hypotheses can be proposed explaining the interplay of zonulin and glioblastoma: elevated zonulin levels (I) contribute to glioblastoma aggressiveness, (II) they mechanistically increase the permeability of the microbiota–gut–brain axis in glioblastoma, and (III) they contribute significantly to a tumorigenic microenvironment and the impairment of the host to eliminate tumor cells. 

### 4.1. (I) Zonulin and Its Mature Form Haptoglobin Produced in Glioblastoma Reduce Prognostic Outcome

Zonulin that shapes glioblastoma is not only provided by peripheral tissues, but the tumor itself can also produce zonulin. An in vitro experiment showed that zonulin is produced by three different glioblastoma cell lines. Zonulin was upregulated when the cell lines were cultured in specific conditions for glioma stem cells [9]. Increased zonulin expression in both tumor tissue and the blood-stream of glioblastoma patients correlates with aggressiveness and worse prognosis measured by a short progression-free survival (PFS) [9]. Elevated levels of zonulin correspond to the malignancy of glial tumors [79]. The correlation of zonulin expression and patients’ survival suggests zonulin as a specific prognostic biomarker for glioblastoma patients [80]. Pre-haptoglobin, which actually corresponds to zonulin, was already proposed as a possible glioblastoma biomarker [80,81,82,83] because it is detected only in glioblastoma plasma but not in the plasma of healthy controls or other oncologic patients (e.g., patients with colon cancer) [80]. In summary, glioblastoma-induced zonulin shapes the malignancy of the tumor.

**Figure 2:**
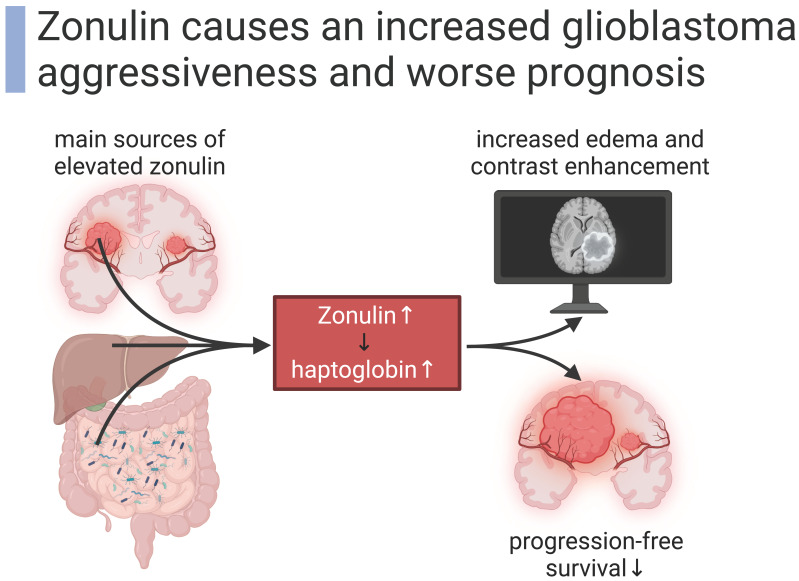
Zonulin induces glioblastoma aggressiveness [9,27,28]. Created with BioRender.com.

The modulation of zonulin cleavage remains to be fully explored in the context of glioblastoma. However, we can hypothesize that elevated zonulin levels shift to higher levels of its cleaved product haptoglobin as epiphenomenon in the context of a dynamic equilibrium. Haptoglobin is an acute phase protein [81] with increased concentrations in colorectal cancer [84], Prostate cancer [85], epithelial ovarian cancers [86], triple-negative breast cancer [87], acute myocardial infarction [88], and heart failure [89]. While acute phase proteins are expected to be elevated in stressful metabolic situations, haptoglobin reaches its highest levels in glioblastoma compared to acute phase reactions (not related to cancer) or other cancer [80,90,91]. Being a cleaved and reassembled form of zonulin, haptoglobin also corresponds to therapeutic advances in glioblastoma patients with significantly decreased serum levels after adjuvant therapy [92]. The processed haptoglobin is expressed in different glioma cell lines in vitro, but the failure to detect haptoglobin protein in the cytosol or serum indicates its transient expression or the need of certain stimuli for haptoglobin to be translated [80,93]. Stimuli for this release of the processed haptoglobin include amyloid-β peptides in Alzheimer’s disease [94], signals secreted in the surrounding of glial tumors [93], or supposedly elevated zonulin levels. Taken together, glial tumors produce elevated levels of zonulin as well as its downstream product haptoglobin, which correlate with clinical severity (Figure 2). 

### 4.2. (II) Zonulin Weakens the Blood–Brain Barrier and the Gut Immune Barrier in Glioblastoma

In glioblastoma tissue, zonulin is expressed in a disrupted BBB and colocalizes with Griffonia simplicifolia lectin (GSI), a marker for endothelial cells [79]. Radiologically, higher levels of zonulin are also associated with an increased edema and contrast enhancement in glioblastoma patients, confirming the disrupted BBB [9]. Conditioned media from glioma cells containing high amounts of zonulin make the endothelial cell layer leaky when compared to conditioned media from healthy astrocytes; neuronal stem cells are attracted to brain pathology, thus transmigrating across the BBB and the trans-endothelial electric resistance of the BBB is decreased [30]. An elimination of zonulin from this glioma conditioned media reduces the number of transmigrating neuronal stem cells and completely reverses the decrease in trans-endothelial electric resistance [30]. Similarly, zonulins procaryotic analog ZOT leads to a higher permeability of bovine brain endothelial cells. ZOT has been proposed as a therapeutic agent to increase the delivery of anticancer agents to the brain [95]. Zonulin as well as its analog ZOT induce leakiness of the BBB.

Claudin-5 is a main component of brain endothelial tight junctions [96]. Both zonulin and claudin-5 locate to the boarders of blood vessels in mice gliomas [30,97]. Elevated zonulin levels lead to decreased expression of claudin-5 [30]. In accordance with the essential role of claudin-5 in preserving the integrity of the BBB, a decrease in claudin-5 expression increases BBB permeability, abnormal neoangiogenesis, and progression in glioblastomas [98]. Altered claudin expression is also associated with the control of cell and organ proliferation and thus influences tumorigenesis. Claudin-1 translocation to the nucleus shapes metastatic behavior in colon cancer [26,99]. The complete absence of claudin-5 leads to 100% mortality of newborn mice, highlighting its indispensable function [100]. Taken together, zonulin prevents claudin-5 from maintaining a functional blood–brain axis, serving as a putative mechanism in glioblastoma carcinogenesis. 

**Figure 3:**
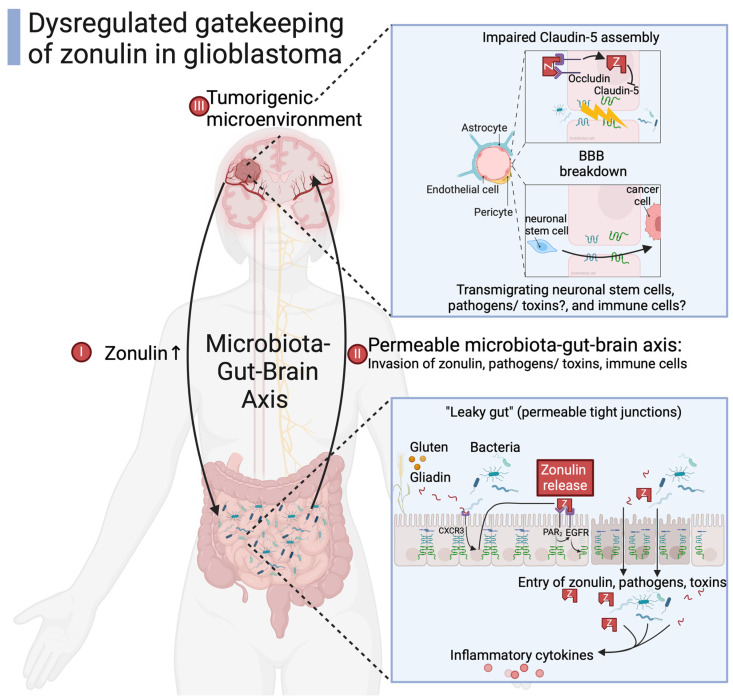
Zonulin as dysregulated gatekeeper of the microbiota–gut–brain axis in glioblastoma [9,10,13,14,23,30,34,42,73]. Created with BioRender.com.

Zonulin does not only reduce claudin-5 expression at the BBB but also at the intestinal (entodermal-) epithelium [10], leading to an impaired gut immune barrier (GIB). The GIB is the hosts’ initial checkpoint against invading microbiota and food antigens, with a tight balance between tolerance and first line of defense [29]. The GIB keeps track of intestinal homeostasis, avoiding chronic inflammation but remaining responsive to acute infectious stressors. A defective GIB may contribute to intestinal inflammation and an invasion of microbiota. Due to the microbiota–gut–brain axis, a deficient GIB also influences brain pathology. In summary, the mutagenic and tumorigenic potential of elevated zonulin levels in glioblastoma could be partly mediated by an increased invasion of microbiota as inflammatory stressors across the impaired GIB and BBB (Figure 3). 

### 4.3. (III) Immune Cells and Microbiota Cross the Disrupted Blood–Brain Barrier, Influencing the Tumorigenic Microenvironment in a Glioblastoma-Favorable Manner

While microglia are the main cells exerting immune functions within the CNS under physiological conditions, upregulated zonulin can grant access to immune cells from the hematopoietic system which are normally absent in the CNS [72]. Whether these invading immune cells potentially fight against the tumor or immediately come under the immunosuppressive control of the tumor remains to be clarified. The leaky BBB, through elevated zonulin levels, disrupts the balanced immune surveillance of the CNS and entails the risk of a chronic inflammatory state.

Besides a higher exposure to immune cells, an increased number of invading microbiota across the impaired gut immune barrier also fosters an inflammatory state with elevated proliferative signaling [58,72]. The gut–brain axis is more precisely named the microbiota–gut–brain axis. This concept emphasizes a short-term inflammatory interaction as well as a long-term genetic co-evolution of microbiota and the host brain [101]. As environmental stressors, bacteria exert selective pressure on agents involved in host–pathogen interaction [102,103,104]. Evolutionary adaptation in the evolutionary arms race between the host and pathogens can help the host to fight invading pathogens. However, these adjustments during evolution can also entail adverse mutations or altered gene expression relevant for emerging tumor growth. The high inter- and intraspecies variability and genetic evolution of zonulin hint at its adaptation during this evolutionary arms race [14]. Zonulin could have initially served as key that opens the BBB for immune cells to launch an early immune response against CNS infections. In the long run, a chronically disrupted BBB with invading neuronal stem cells, immune cells, and pathogens/toxins could exert selective pressure on mutagenesis in the CNS and could therefore be partly responsible for emerging glial tumors.

The zonulin pathway is finely tuned and achieves a disassembly of tight junctions within few hours and is mostly reversible afterwards. Nevertheless, zonulin also causes chronic effects on the BBB, evident by an impaired claudin-5 assembly in the long run [10,29]. More permeable barriers through zonulin permit the immune system to launch an early immune response against pathogens and allergens, but a chronic state of inflammation also entails a risk of DNA damage and tumor development. Chronically elevated zonulin levels might shape tumor development: the incidence of glioblastoma increases in the elderly, with a median age at diagnosis of 66 years [1], and zonulin levels correlate with age-related cytokines TNF-α and IL-6 and gut permeability [105]. The increased exposure of the CNS to inflammatory stressors can help the tumor to acquire capabilities such as (A) tissue invasion with promotion of growth signals and increased cellular replication, (B) evasion of apoptosis and anti-growth signals, and (C) angiogenesis [106].

**Figure 4:**
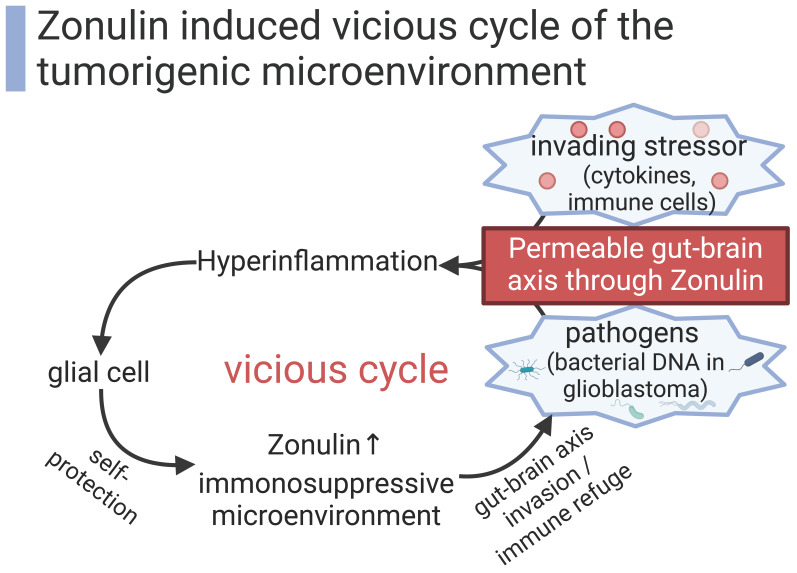
Elevated zonulin levels create a carcinogenic environment in a vicious cycle [9,42,43,55]. Created with BioRender.com.

Besides its disruption of the BBB with indirect changes in the intracerebral immune surveillance, zonulin has also been shown to directly modulate the immune response. Zonulin concentration correlates with higher numbers of neutrophils, functional activity of circulating neutrophils, higher numbers of CD3^+^CD8^+^ and NK cells, and lower CD19^+^ cells in blood samples of patients with inflammatory bowel disease [107]. Besides cellular immunity, zonulin levels also correlate with serum IL10, IL17, and IL22 in patients with inflammatory bowel disease [108] and with IL6, TNF-α, and lipopolysaccharide in Vitiligo patients [109]. Moreover, the zonulin antagonist *Larazotide* has been shown to alter T cell homeostasis in the spleen with a shift towards anti-inflammatory signaling in a model of arthritis [110]. Taken together, zonulin could enhance the local immunosuppression already established by glioblastomas in a vicious cycle (Figure 4). 

## 5. Future Perspectives

Future research is needed to fill the controversies and gaps in knowledge regarding the function of zonulin in glial tumors. The role of zonulin in CNS carcinogenesis needs to be further investigated to improve the prognostic outcome of glioblastoma patients. We propose three ideas to take advantage of zonulin: (I) as a prognostic biomarker, (II) as a therapeutic modulator of tumor progression, and (III) as a target for drug delivery across the BBB.

In particular, relapse rates are extremely high in glioblastoma patients, so close surveillance is necessary to enable early therapeutic intervention. We are currently facing two major difficulties regarding the monitoring of glioblastoma progression. Firstly, imaging modalities may not clearly differentiate between tumor progression and treatment-related “pseudo-progression” in the early phase of follow-up after surgery/radio–chemotherapy [111]. Secondly, tissue biopsies have limitations such as diagnostic follow-up regarding clinical complications, intricate accessibility, and failure to represent the heterogeneity of the whole tumor [112]. Therefore, biomarkers that can be easily assessed in liquid biopsies especially in minimally invasive blood samples are necessary for “real-time” evaluation of tumor growth and therapeutic efficacy [112]. The prognostic potential of zonulin in glioblastoma patients has been investigated with promising data [80,81,82,83]. These findings need to be confirmed in bigger cohorts and regarding the response to treatment or relapse.

Zonulin could also improve prognostic outcomes of glioblastoma patients as therapeutic targets. The modulation of different agents of the zonulin pathway has already been proven successful in protecting the BBB [113,114,115]. Therefore, an inhibition of zonulin itself and all its associated putatively pro-tumoral effects in glioblastoma seem to be reasonable to prevent BBB disruption. The zonulin antagonist Larazotide (also named AT-1001 or FZI/0) reduces antigen and immune cell trafficking across tight junctions [116,117] through a blockade of the zonulin receptor without an alteration of zonulin levels [118,119]. Several clinical studies have already shown the good tolerability and promising therapeutic effects of *Larazotide* on Celiac disease and COVID-19-MIS-C (Multisystem Inflammatory Syndrome in Children) [116,120]. *Larazotide* still remains to be investigated in CNS diseases: *Larazotide* needs to be further modified or administered intravenously/intrathecally to achieve its effects on the CNS [121] because it was originally used only in Celiac disease with intestinal target [122]. Nutritional changes with reduced wheat intake and thus decreased gliadin, as one of the main stimulators for zonulin release, could also modulate zonulin levels. Moreover, changes in the gut microbiome, potentially mediated by altered zonulin levels, may influence the immune environment in glioblastoma and the efficacy of chemotherapeutic treatments [74,123,124,125]. This raises the question as to what extent nutrition influences microbiota and zonulin levels and how both shape glioblastoma. For example, a probiotic treatment reduces serum zonulin and postoperative infectious complications in patients with colorectal liver metastases [126]. Similarly, *Lactobacillus* has protective effects on the epithelial barrier through reduced zonulin release [37]. The therapeutic applicability of protective microbiota targeting zonulin levels and glioblastoma prognosis needs further investigation regarding optimal pro- and prebiotics. In summary, zonulin antagonists, a modification of gliadin, and nutrition could serve as therapeutic options to influence zonulin-mediated glioblastoma progression.

Finally, effective drugs against glioblastoma require sufficient access to the CNS across the BBB [3,127]. With only a handful of FDA-approved therapies within the last 30 years, it has been proposed that the failure of drug treatment in glioblastoma patients is due to reduced and heterogenous drug delivery across the BBB [40]. The zonulin receptor agonist AT-1002 is already used in drug development for other diseases to increase absorption by opening tight junctions [128]. Already existing therapeutic options could be co-administered with a single-shot dosage of zonulin agonists. Therefore, one of the capacities of zonulin (i.e., BBB disruption) can be its use for more effective drug delivery across the BBB in glioblastoma patients and also putatively in patients with brain metastases.

## 6. Conclusions

Elevated zonulin levels have been associated with worse prognoses of glioblastoma patients. Higher tumor aggressiveness can be explained mechanistically by a disrupted BBB and a permeable gut immune barrier. Increased invasion of microbiota and immune cells leads to hyperinflammation and reduced immune response against glial tumor cells. The tumorigenic microenvironment grows out of control of the immune system and the rising zonulin levels result in a vicious cycle. If these hypotheses are confirmed in further clinical studies, zonulin could be used as a prognostic biomarker, and the modulation of the zonulin pathway could serve as a therapeutic option in patients with glioblastoma and even other brain tumors. Zonulin is a promising causal, diagnostic, and therapeutic agent in CNS neoplasms with many unresolved questions that should be explored in the near future.

## Data Availability

Any data related to the work are available upon request.
